# FATP1-mediated fatty acid uptake in renal tubular cells as a countermeasure for hypothermia

**DOI:** 10.1007/s00109-025-02525-0

**Published:** 2025-03-05

**Authors:** Kie Horioka, Hiroki Tanaka, Shimpei Watanabe, Shinnosuke Yamada, Shuhei Takauji, Akira Hayakawa, Shotaro Isozaki, Keisuke Okaba, Namiko Ishii, Ayumi Motomura, Hiroyuki Inoue, Lynda Addo, Daisuke Yajima, Yoichiro Takahashi, Henrik Druid, Lasse Pakanen, Katja Porvari

**Affiliations:** 1 Department of Forensic Medicine, Research Unit of Biomedicine and Internal Medicine, Medical Research Center Oulu, University of Oulu, Oulu, Finland; 2 Department of Legal Medicine, Institute of Medicine, University of Tsukuba, Tsukuba, Japan; 3 Department of Oncology-Pathology, Karolinska Institutet, Stockholm, Sweden; 4 Department of Legal Medicine, International University of Health and Welfare, Narita, Japan; 5 Division of Tumor Pathology, Department of Pathology, Asahikawa Medical University, Asahikawa, Japan; 6 Forensic Science Group, RIKEN SPring-8 Center, Sayo, Japan; 7 Department of Anatomy, International University of Health and Welfare, Narita, Japan; 8 Department of Emergency Medicine, Hokkaido University, Sapporo, Japan; 9 Department of Forensic Sciences, Akita University Graduate School of Medicine, Akita, Japan; 10 Department of Forensic Medicine, Tokai University School of Medicine, Isehara, Japan; 11 Department of Medical Laboratory Technology, Accra Technical University, Accra, Ghana; 12 Forensic Medicine Unit, Finnish Institute for Health and Welfare (THL), Oulu, Finland

**Keywords:** Hypothermia, Renal tubular cells, Fatty acid, β-Oxidation, FATP1

## Abstract

**Abstract:**

Hypothermia is a condition in which body temperature falls below 35 °C, resulting from exposure to low environmental temperatures or underlying medical conditions. Postmortem examinations have revealed increased levels of fatty acids in blood and lipid droplet formation in renal tubules during hypothermia. However, the causes and implications of these findings are unclear. This study aimed to analyze the biological significance of these phenomena through lipidomics and transcriptomics analyses of specimens from emergency hypothermia patients and mouse hypothermia models. Both human hypothermia patients and murine models exhibited elevated plasma concentrations of fatty acids and their derivatives compared with controls. Hypothermic mouse kidneys displayed lipid droplet formation, with gene expression analysis revealing enhanced fatty acid uptake and β-oxidation in renal tubular cells. In primary cultured mouse renal proximal tubular cells, low temperatures increased the expression levels of Fatty acid transport protein 1 (FATP1), a fatty acid transporter, and boosted oxygen consumption via β-oxidation. Mice treated with FATP1 inhibitors showed a more rapid decrease in body temperature upon exposure to low temperatures compared with untreated mice. In conclusion, increased fatty acid uptake mediated by FATP1 in renal tubular cells plays a protective role during hypothermia.

**Key messages:**

- Low temperatures increase FATP1 expression and fatty acid uptake in renal proximal tubular cells, resulting in enhanced β-oxidation.
- Renal proximal tubular cells play an important role in the resistance to hypothermia via lipid uptake.
- Maintaining renal lipid metabolism is essential for cold stress adaptation.

**Supplementary Information:**

The online version contains supplementary material available at 10.1007/s00109-025-02525-0.

## Introduction

Accidental hypothermia is an emergency in which the body temperature decreases to below 35 °C. It is caused by prolonged exposure to low ambient temperature resulting in a drop in body temperature at a higher rate than that of heat generation [1–5]. Accidental hypothermia is defined as an involuntary drop in core body temperature below 35 °C and can occur even in healthy individuals without underlying disease [6]. Such patients often require emergency treatment and may experience complications such as coma and arrhythmia [7–9]. Mild hypothermia (32 to 35 °C) can be used as a protective therapy for medical purposes. This treatment can ease the damage to the brain after the resuscitation of patients in cardiac arrest [10–13]. In hibernating species, hypothermia is a biological phenomenon that helps to protect the functions of the various organs [14]. We have previously reported that the activation of splenic platelets in hypothermic mice helps to restore skeletal muscle damage that occurs by shivering [15]. These reports indicate that various biological reactions occur during hypothermia to protect tissues and ultimately maintain life. However, hypothermia has also been reported to stress the organism and cause dysfunction [16], suggesting that the physiological response to sustain life in a low-temperature environment can sometimes result in adverse effects. Although an organism would be expected to suffer problems from exposure to extreme environmental temperatures, upon closer consideration, the symptoms appear as a complex phenotype. This involves cellular damage in the various organs directly affected by the low temperatures, as well as resistance responses in some organs, making the pathophysiology of hypothermia difficult to understand.

Several forensic research groups have reported elevated blood levels of free fatty acids (FFAs) to be a hallmark of accidental hypothermia [17, 18]. These increased levels in fatal hypothermia cases are thought to result from the sympathetic-adrenal system and increased lipolysis in adipose tissue, including both brown adipose tissue (BAT) and white adipose tissue (WAT), to maintain thermal homeostasis [19, 20]. Furthermore, vacuolizations of renal tubular epithelial cells have been reported in accidental hypothermia [21, 22]. Because these vacuoles can be stained with Oil Red O, they are believed to have lipid component accumulation. Renal proximal tubular cells are highly differentiated cells and, under physiological conditions, require significant amounts of energy to take up a variety of molecules, including glucose, amino acids, and ions reabsorbed from primary urine [23–27]. Lipid droplets in renal tubules have been observed in forensic autopsy cases with renal alterations, such as deaths caused by starvation, severe alcohol intoxication, and ketoacidosis associated with diabetes mellitus [24, 28, 29]. Under normal conditions, the kidneys should not store lipids unless there is an underlying pathological condition of chronically high blood lipid concentrations. The high FFA levels during hypothermia are thought to induce renal tissue lipid uptake as an energy source, leading to lipid droplet formation. However, because such rapid increases in blood FFA levels rarely occur, it is currently unclear whether this phenomenon is beneficial or detrimental to the human body.

The primary objective of this study was to analyze the effects of lipid droplet formation on renal function during hypothermia. Here, samples from hypothermic patients and mouse models of hypothermia were analyzed using lipidomics and RNA sequencing (RNA-seq) to comprehensively explore the molecular landscape associated with renal lipid deposition under hypothermic conditions.

## Materials and methods

### Clinical samples

Blood test data and clinical information from 451 hypothermia patients treated in the multicenter emergency care study in Japan (ICE-CRASH study) [7] were analyzed. Plasma samples prepared using sodium citrate as the anticoagulant and collected at the time of emergency transport from 25 of these cases with no background diabetes or other metabolic disease and with obvious environmental cold exposure were used for plasma FFA analysis and lipidomics (Supplemental Table 1). Twenty-five commercially available normal plasma samples (George King Bio-Medical, Inc., Overland Park, KS, USA) were used as comparators for the analysis. Clinical data on hypothermia are shown in Supplemental Table 2.

### Plasma lipidomics

One volume of mouse or human plasma was added to two volumes of acetonitrile/ethanol extraction buffer (9:1, containing 0.075% formic acid), vortexed for 10 min, and centrifuged at 1000 × *g* for 10 min. Then, the supernatant was collected and analyzed on an Orbitrap LC–MS system (Thermo Fisher Scientific, Waltham, MA, USA). Separation was performed on a reverse phase C18 column at 40 °C and 0.5 mL/min flow. We acquired spectrometric data in the 100–600 m/*z* range both in negative and positive polarity. The raw data were then processed, and each metabolite was quantified using MS-DIAL (RIKEN CSRS/IMS, Yokohama, Japan).

### Enzymatic analysis of FFA and triglyceride (TG)

The quantitative assessment of total FFAs and total TGs in plasma and tissue samples was performed using LabAssay NEFA (Fujifilm Wako, Tokyo, Japan) and LabAssay TG (Fujifilm Wako), respectively. Kidney tissues were crushed and lysed in PBS containing 0.1% NP40 to a 10 mg/mL wet weight, then the supernatant was used for analysis.

### Animal model

We used 8-week-old male C57Bl/6 mice for the hypothermia model (Hyp). In the hypothermia model, mice were kept in aluminum cages without floor coverings at an ambient temperature of 4 °C and their rectal temperature was measured every 2 h by electronic thermometer. Our previous studies have shown that rewarming therapy can restore the mice to an active status, even after lowering their rectal temperature to 15 °C [30]. In the present study, blood and kidney samples were taken under isoflurane inhalation anesthesia, defined as the humanitarian endpoint after confirming the maintenance of cardiopulmonary function when the rectal temperature was reduced to 15 °C. The FATP1-suppressed mouse model was treated by gavage with FATP1-in-1 (CAS no. 1431945–95-7) at a dose of 20 mg/kg, followed by the same treatment as described above. Comparison subjects were also kept at room temperature after administration of FATP1-in-1. The whole blood was mixed with 3.2% sodium citrate solution in a 9:1 ratio for plasma isolation.

### Histopathological analysis and imaging analysis

For histopathological analysis of kidney tissues, formalin-fixed paraffin-embedded specimens were prepared. In bright-field microscopy, slides were stained using standard protocols for H&E (Mayer’s Hematoxylin and Eosin, Muto Chemical, Tokyo, Japan). The kidneys were immersed in 4% PFA/PBS overnight, followed by immersion in 30% sucrose. Subsequently, the kidney tissue was then frozen using Presto CHILL (Milestone, Valbrembo, Italy), and frozen sections were cut at a thickness of 7 μm using a Leica CM3050 S (Leica, Deer Park, IL, United States). Lipid droplets in renal tubular cells were analyzed by Oil Red O staining and fluorescence staining with BODIPY (Thermo Fisher Scientific). For the immunohistochemical analysis, the sections were sequentially treated in the following way: deparaffinization, rehydration, endogenous peroxidase quenching, and antigen retrieval with Tris/EDTA buffer, pH9.0 (AgilentDako, Santa Clara, United States). The sections were incubated with primary antibodies (Supplemental Table 3), followed by detection with an anti-mouse or anti-rabbit horseradish peroxidase (HRP)-conjugated secondary antibody (Vector Labs, Burlingame, CA, USA). Lotus tetragonolobus lectin (LTL; Thermo Fisher Scientific) was used as a marker for proximal tubular cells for the immunofluorescence staining with Peroxisome proliferator-activated receptor α (PPARα), FATP1, and Perilipin 5 (PLIN5). Mouse renal proximal tubular epithelial cells (MRPTEpiC) were grown on collagen plates and used for fluorescence staining with BODIPY or FATP1 antibody. The BODIPY-positive area in the proximal renal tubular cells was quantified, with representative images of each sample taken under × 400 magnification. After binarization of each positively stained area with imaging software WinROOF 2018 (Mitani Corporation, Tokyo, Japan), the numbers of positive lipid droplets and mean area of positive lipid droplets in the cells were calculated. Quantification of the immunohistochemical staining was conducted using WinROOF 2018 by determining the positive area or cell count.

### Transmission electron  microscopy (TEM)

For TEM analysis, the 1 mm^3^ kidney tissue samples were fixed in 2.5% glutaraldehyde and 2% paraformaldehyde in 0.1 mol/L phosphate buffer, followed by fixation in 1% osmium tetroxide for 1 h at 4 °C. The samples were then processed via sequential alcohol dehydration and further processed for Epon embedding. For tissue examination, semi-thin sections were cut and stained with toluidine blue. Ultra-thin sections were cut and stained with platinum blue (TI blue, Nisshin EM, Tokyo, Japan) and Reynolds’ lead citrate. Finally, the sections were imaged using TEM (JEOL JEM-1200EX2).

### Mass spectrometry imaging (MSI)

Frozen sections of kidney tissues were prepared from the control and hypothermia groups. These were mounted onto conductive indium tin oxide (ITO)-coated slides. MSI analyses were performed with a Synapt XS high-resolution mass spectrometer equipped with a two-dimensional Desorption Electrospray Ionization (Waters Xevo TQ-XS). The DESI parameters were as follows: capillary voltage: 4.0 kV, cone voltage: 50, source temperature: 1300 °C, spray solvent: 3 µL/min (98% MeOH), mode: negative, *m*/*z*: 100–1200, pixel size: 100 µm, N2 gas flow: 0.5 MPa, section: 10 µm.

### RNA-seq

Total RNA was extracted from kidney tissues collected from five control mice and five hypothermia mice using a RNeasy mini kit (Qiagen, Hilden, Germany). MRPTEpiC was incubated at 37 °C (control) or 28 °C (hypothermia) for 5 h, then RNA was extracted using the same method. The sequencing data were obtained by NovaSeq (Illumina, San Diego, CA, USA). Reads mapping, read counts, and gene expression analysis were performed by CLC Genomic workbench (Qiagen). Each gene expression level was calculated as a TPM (transcripts per million), and those with *P*-value < 0.05 were extracted and used for GSEA.

### Western blot analysis

Protein samples were separated using SDS-PAGE and electrotransferred to nitrocellulose membranes. The membranes were then probed with primary antibodies against PPARα, FATP1, PLIN5, Carnitine palmitoyltransferase 1A (CPT1), CD36, and β-Actin (Supplemental Table 3). The membranes were incubated with the respective HRP-conjugated anti-rabbit or anti-mouse IgG secondary antibodies (R&D Systems, Minneapolis, MN, USA). Antibody binding was visualized using the SuperSignal West Pico Chemiluminescent Substrate (Thermo Fisher Scientific) and Azure imager (Azure biosystems, CA, USA). Quantification was performed by densitometry analysis using Image J software (NIH, Bethesda, MD, USA).

### Cell culture

Mouse renal proximal tubular epithelial cells (MRPTEpiCs) were purchased from Innoprot (Bizkaia, Spain). These cells were cultured in Epithelial Cell Medium containing essential and non-essential amino acids, vitamins, organic and inorganic compounds, hormones, growth factors, trace minerals, and 2% fetal bovine serum. RNA and proteins were extracted from these cells for gene expression analysis. To analyze FFA uptake into MRPTEpiCs under low temperature conditions, cells were seeded into plates, incubated at low temperature in the culture medium supplemented with 0.1% BSA and 1 mM PA, fixed with formalin, and stained with BODIPY (Thermo Fisher Scientific), then microscopically observed and quantified using a fluorescent plate reader. To analyze the fatty acid oxidization activity in MRPTEpiCs, Seahorse flex analysis (Agilent, Santa Clara, CA, USA) was performed. Oxygen consumption of cells with and without PA was measured over time at 37 °C or 28 °C. The damage and viability of MRPTEpiCs under low temperature conditions were analyzed by LDH Cytotoxicity analysis and Sulforhodamine B (SRB) analysis, respectively.

### Real-time quantitative polymerase chain reaction (qRT-PCR)

Total RNA was isolated from MRPTEpiC, and qRT-PCR was performed using one-step real-time RT-PCR (Qiagen). To analyze the expression levels of mouse *Slc27a1* mRNA, the following primer sets were used: forward primer: 5′-tgcttttttctgggactt-3′, reverse primer: 5′-gctctagccgaacgaatc-3′. The expression levels of mouse *Actb* mRNA were also analyzed as an internal control with the following primer set: forward primer: 5′-ctgtattcccctcatcgtg-3′, reverse primer: 5′-ctctcatgtcgtcccagt-3′. Each cycle threshold (CT) value was obtained, then the 2^−ΔΔCT^ method was used to calculate the relative expression values.

### Statistical analysis

GraphPad Prism 10 (GraphPad Software, San Diego, CA, USA) was used for all statistical analyses. The differences in the experimental values were analyzed via the Mann–Whitney *U* test, one-way ANOVA followed by the Tukey–Kramer method, and Log-rank test. *P*-values < 0.05 were considered statistically significant.

## Results

### Hypothermia increases blood FFA levels

**Fig. 1:**
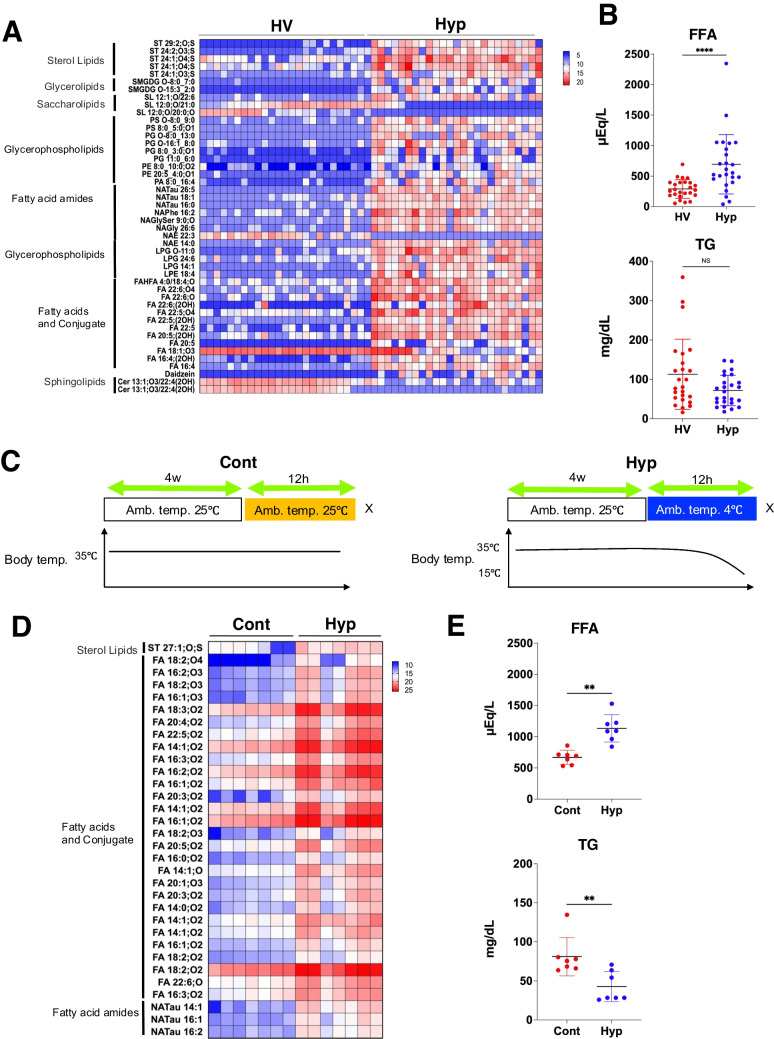
Quantitative evaluation of blood free fatty acids (FFAs) and triglycerides (TGs) in hypothermia patients and mouse hypothermia models. **A** Lipidomics analysis of plasma samples obtained from hypothermia patients. Heatmaps showing lipids with observed changes in log2 fold change (FC) < 5 and > 5 comparing the healthy volunteers and accidental hypothermia patients (*n* = 25 each). Data are shown for the MS analysis in negative mode (measurement results in positive mode are shown in Supplemental Fig. 1A). **B** Plasma total FFA and TG concentrations analyzed by the enzymatic method in healthy volunteers and accidental hypothermia groups (*n* = 25 each). **C** Schematic illustration of the mouse model of hypothermia. **D** Lipidomics in plasma samples obtained from hypothermic mice. Heatmaps showing lipids with observed changes in log2 FC, <  − 3 and > 3 comparing the control mice and hypothermic mice (*n* = 7 each). Data are shown for the MS analysis in negative mode (measurement results in positive mode are shown in Supplemental Fig. 1B). **E** Plasma total FFA and TG concentrations analyzed by the enzymatic method in control and hypothermic mice (*n* = 7 each). Data were analyzed by the Mann–Whitney *U* test. All data are represented as the mean ± standard error of the mean. **P < 0.01, ****P < 0.0001. HV: healthy volunteer group; Hyp: accidental hypothermia group; Cont: control group; Hyp: hypothermia group

The FFA concentrations in blood samples collected at forensic autopsies of hypothermia cases are reportedly higher than those of non-hypothermia cases [17]. However, it is not clear how this finding translates to the clinical situation, since postmortem changes in the metabolome may impact on the levels of smaller molecules such as FFA. Therefore, we analyzed plasma FFA levels in samples from individuals who were brought to the emergency room with hypothermia to determine if increased FFA levels could be observed in living hypothermic patients. We used lipidomics with liquid chromatography-high-resolution mass spectrometry (LC-HRMS) to examine the lipid dynamics in plasma samples from 25 healthy volunteers (HV) and 25 hypothermia patients (Hyp) who had no background diabetes or other metabolic diseases and had obvious cold exposure. The results suggested that compared with the normal volunteers, the hypothermic patients exhibited a higher abundance of plasma FFAs, along with their metabolites and intermediates involved in the synthesis of FFAs to TGs (Fig. 1A). The total FFA and TG levels in these blood samples were measured using the enzyme-chemical chromogenic assay. The total of FFAs was clearly higher in hypothermic patients than in healthy volunteers. Although the total of TGs was similar between the groups, it tended to be slightly lower in hypothermic patients compared with healthy volunteers (Fig. 1B). To perform a more detailed analysis of this phenomenon, samples from murine models of hypothermia (Fig. 1C), which mimic human hypothermia, were used. Lipidomics of hypothermic mouse plasma samples detected more FFAs of various lengths from 14 to 22 carbons than in the normal control samples (Fig. 1D). Analysis of FFAs and TGs with the enzyme-chemical chromogenic assay suggested higher levels of FFAs and lower levels of TGs in the hypothermic mice compared with the controls (Fig. 1E). These results indicate an increased abundance of FFAs and conjugated FFAs identified by mass spectrometry in both human accidental hypothermia cases and mouse models of hypothermia.

### Lipid droplet formation in kidney tissues in hypothermia

**Fig. 2:**
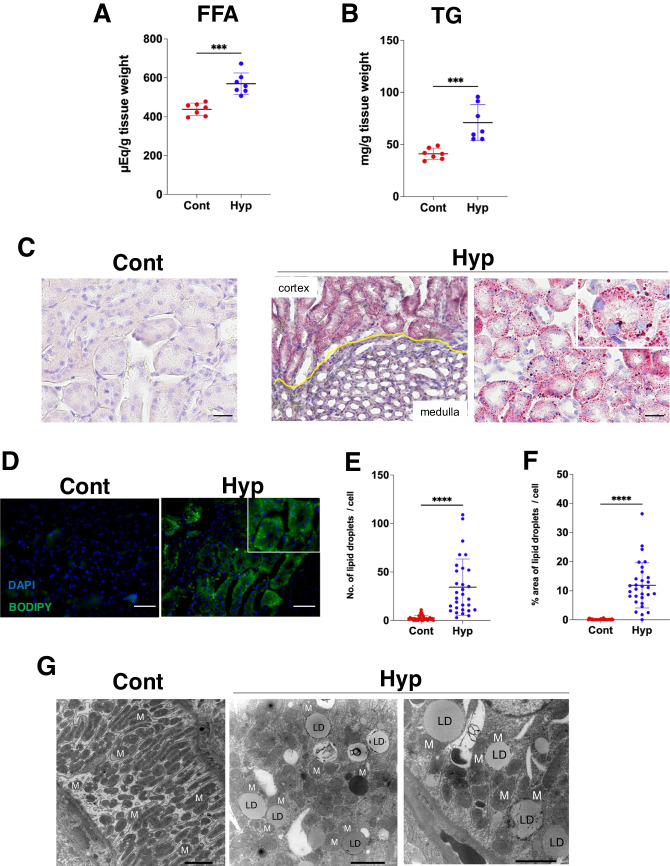
Lipid droplet formation in renal proximal tubules. **A** Free fatty acid (FFA) levels and **B** triglyceride (TG) levels in renal tissue extracts (*n* = 7, respectively). **C** Oil red O staining and **D** BODIPY staining in control mice and hypothermic mice renal tissues. Scale bars: 50 μm. **E** Number of lipid droplets per cell in Oil red O staining (*n* = 5). **F** % area of lipid droplets per cell in Oil red O staining. **G** Transmission electron microscopy analysis of hypothermia model mice tissues. The mitochondria (M) are observed along the periphery of the lipid droplets (LD). Scale bar: 2 µm. Data were analyzed by one-way ANOVA followed by the Mann–Whitney *U* test. All data are represented as the mean ± standard error of the mean. ***P < 0.001, ****P < 0.0001. Cont: control group; Hyp: hypothermia group

Enzymatic methods were used to examine the FFA and TG levels in renal tissue extracts. The results showed that both FFAs and TGs were higher in renal tissues from hypothermic mice than in renal tissues from the normal control mice (Fig. 2A, B). Oil Red O staining of kidney tissue sections showed no detectable staining in normal mouse samples, but prominent lipid droplets were observed in the renal proximal tubular cells of hypothermic mice (Fig. 2C). In addition, fluorescent observation with BODIPY staining revealed that lipid droplets accumulated mainly at the basolateral side of the proximal tubules (Fig. 2D). The number and % area per cell of these lipid droplets were significantly higher in the hypothermic mice (Fig. 2E, F). TEM analysis also revealed numerous lipid droplets in the proximal tubule cells of the hypothermic mice (Fig. 2G). Further examination of these lipid droplets suggested that the route of intracellular lipid entry was from the capillary side rather than from the primary urine. Therefore, these findings suggest that there is a significant increase in the lipid content of tubular epithelial cells.

### FFAs and TGs are predominantly stored in the renal cortex

**Fig. 3:**
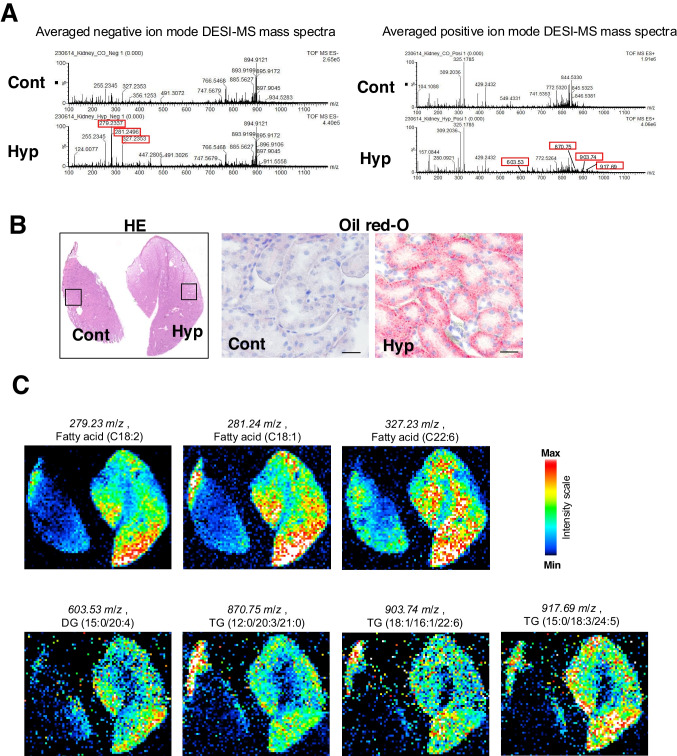
Mass spectrometry imaging analysisin hypothermic mice.** A** The averaged negative and positive ion mode analyses of the DESI-MS spectrum in the renal tissue. **B** Hematoxylin and eosin (H&E) staining and Oil red O staining images of the renal tissues analyzed by DESI-MS spectrometry. Scale bars: 50 μm. **C** Representative ion images in negative and positive mode of renal tissues from control and hypothermic mice. Cont: control group; Hyp: hypothermia group

MSI was then used to examine the lipid distribution in the kidneys of the hypothermic mice. When analyzed in negative ion mode, lipids with *m/z* values of 279.2337, 281.2496, and 327.2353 were detected more than twice as abundantly in hypothermic mice compared with control mice (Fig. 3A, left). When analyzed in positive ion mode, lipids with *m/z* values of 603.53, 870.75, 903.74, and 917.69 were detected more than twice as abundantly in hypothermic mice compared with control mice (Fig. 3A, right). To confirm the lipid accumulation distribution in the renal tubules from hypothermia, MSI was performed on specimens with lipid accumulation in the proximal tubules (Fig. 3B). The *m/z* value of each lipid was cross-referenced with the SWISS lipid database to estimate the type of lipid. The lipids with *m/z* values of 279.23, 281.24, and 327.23 were estimated to be fatty acid (18:2), fatty acid (18:1), and fatty acid (C22:6(n-3); Docosahexaenoic acid [DHA]), respectively. The lipids with *m/z* values of 603.53, 870.75, 903.74, and 917.69 were estimated to be diacylglycerol (15:0/20:4), triacylglycerol (12:0/20:3/21:0), triacylglycerol (18:1/16:1/22:6), and triacylglycerol (15:0/18:3/24:5), respectively. Analysis of their tissue distribution in the hypothermic mice kidneys revealed that the FFAs and their accumulated forms, diacylglycerols (DGs) and TGs, were predominantly found in the cortex, while DHA, which also functions as a signaling molecule, was mainly found in the medulla (Fig. 3C). These results indicated that in hypothermic kidneys, the sources of energy, FFAs and TGs, are stored mostly in the renal cortex, which is rich in proximal tubular cells.

### Hypothermia exhibits a gene expression pattern that accelerates lipid metabolism in the kidney

**Fig. 4:**
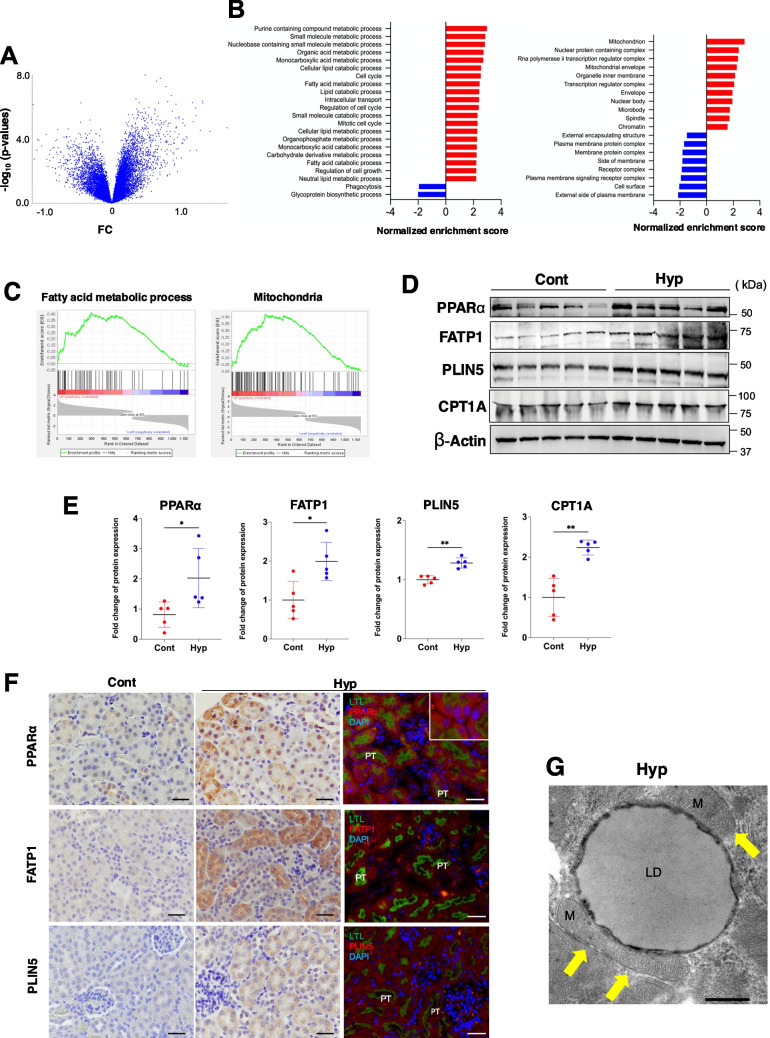
Gene expression analysis of hypothermic mouse renal tissues. **A** Volcano plot of gene expression analysis of control and hypothermic mouse renal tissues (*n* = 5 each). **B** Normalized enrichment score analyzed by gene set enrichment analysis (GSEA). Left: Gene Ontology (GO) biological process; right: GO cellular component. **C** Representative GSEA results. **D** Western blot analysis of PPAR alpha, FATP1, PLIN5, CPT1A, and Actin protein expression in whole kidney lysates of control and hypothermic mice (*n* = 5 each). **E** Quantitative data from PPARα, FATP1, PLIN5, and CPT1A Western blot data (*n* = 5 each). **F** Immunohistochemical and immunofluorescence staining of PPARα, FATP1, and PLIN5 protein expression in renal tissues. Scale bars: 50 μm. **G** Electron microscopy of proximal tubular cells in the hypothermia model. Lipid droplets were observed in contact with the mitochondria (arrow). Scale bars: 500 nm. Data were analyzed by the Mann–Whitney *U* test. All data are represented as the mean ± standard error of the mean. *P < 0.05, **P < 0.01. Cont: control group; Hyp: hypothermia group

To analyze the specific gene expression changes underlying the observed renal tissue lipid accumulation, we performed an RNA-seq analysis on kidney samples from control and hypothermic mice. In this analysis (Fig. 4A), genes with a *P*-value < 0.05 were extracted and subjected to gene set enrichment analysis (GSEA). The GSEA scores are indicated in Fig. 4B. These results revealed transcripts that encode proteins involved in FFA metabolic processes and mitochondria (Fig. 4C and Supplemental Fig. 2A). Overall, genes associated with increased β-oxidation were more abundantly expressed in the hypothermia model compared with the controls, which may correspond to a more efficient energy conversion of FFAs. Notably, these included *Plin5*, *Cpt1a*, *Slc27a1*, and *Ppara* mRNAs. *Plin5* mRNA encodes the protein responsible for converting excess FFAs to TGs and storing them in lipid droplets as a supply to the mitochondria, while *Cpt1a* mRNA encodes the rate-limiting enzymes for β-oxidation. Furthermore, *Slc27a1* mRNA encodes the FATP1 protein, a transporter responsible for FFA cellular uptake. *Ppara* mRNA encodes a master transcription factor for lipid metabolism (Supplemental Fig. 2B). Western blot analysis was performed to analyze the expression levels of the corresponding proteins of these FFA metabolism-related mRNAs. The data showed that the protein expression levels of PPARα, FATP1, PLIN5, and CPT1A were upregulated in the renal tissues of hypothermic mice (Fig. 4D/E). Interestingly, CD36, another well-known plasma membrane protein involved in FFA uptake [31, 32], was not upregulated in the hypothermic mice renal tissues at either the mRNA or protein level (Supplemental Fig. 2C). Immunohistochemistry and immunofluorescence for PPARα, FATP1, and PLIN5, combined with LTL as a proximal tubule marker, revealed strong expression of these proteins in LTL-positive proximal tubules in the hypothermia group (Fig. 4F). We detected PPARα signals in both cytoplasm and nuclear chromatin in immunofluorescence, suggesting that PPARα is transcriptionally active. PLIN5 is a protein expressed on the membrane surface of intracellular lipid droplets and is involved in FFA translocation to the mitochondria [33, 34]. Our TEM analysis of the hypothermic mice renal tissues showed attachment of lipid droplets to the mitochondria (Fig. 4G). These gene expression analyses indicated an enhancement of FFA uptake via FATP1 in the proximal tubules of hypothermic mouse kidneys and that these FFAs were provided to the mitochondria.

### Low temperature increases FFA import and β-oxidation in renal tubular cells

**Fig. 5:**
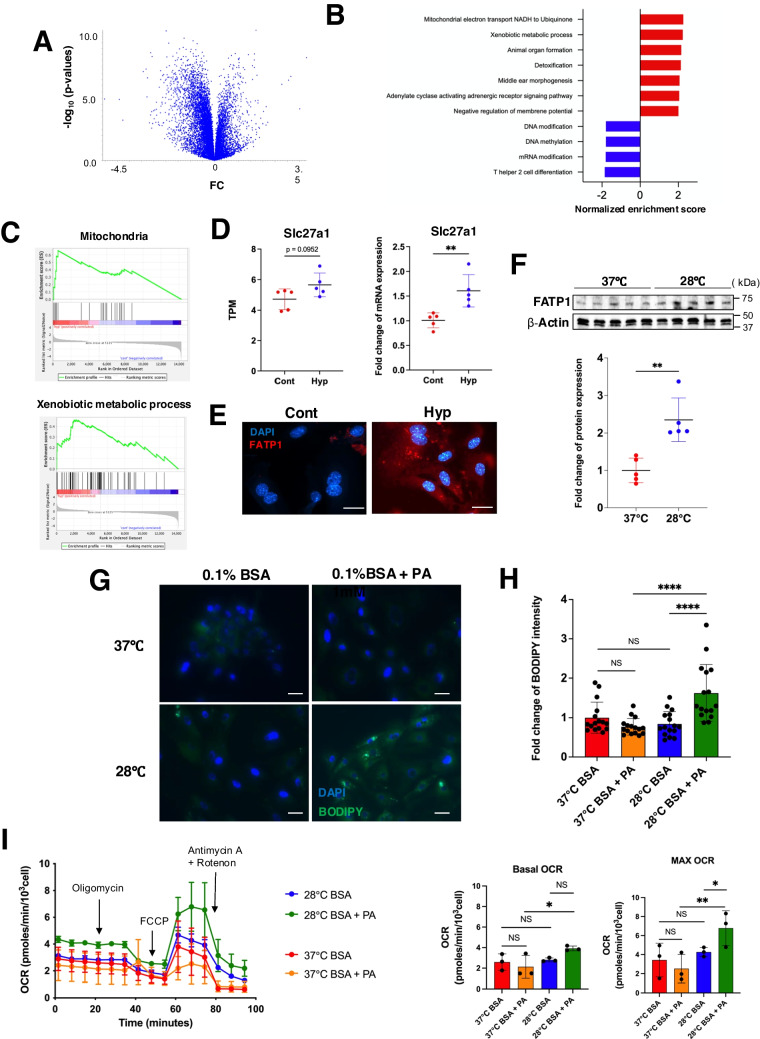
Effect of low temperature on cultured mouse proximal tubular cells.** A** Volcano plot representing the gene expression variation when mouse renal proximal tubular epithelial cells (MRPTEpiCs) were incubated at low temperature (28 °C, *n* = 5) compared with standard culture temperature (37 °C, *n* = 5). **B** Normalized enrichment score analyzed by gene set enrichment analysis (GSEA; Gene Ontology biological process). **C** Representative GSEA results. **D** *Slc27a1* mRNA expression levels in MRPTEpiCs cultured with standard and low temperatures, RNA sequencing data (left) and qRT-PCR data (right) (*n* = 5, respectively). **E** FATP1 protein expression levels in MRPTEpiCs analyzed by immunofluorescence staining. Scale bars: 10 μm. **F** Western blot analysis of FATP1 protein expression in MRPTEpiCs cultured at standard and low temperatures (*n* = 5, respectively). **G** Analysis of lipid accumulation in MRPTEpiCs cultured at standard and low temperatures. BODYPI (green) and DAPI (blue). Scale bars: 10 μm. **H** Quantitative analysis of fluorescence intensity representing accumulated lipids in MRPTEpiCs cultured at standard and low temperatures (*n* = 16 each). **I** Oxygen consumption rate (OCR) determined for MRPTEpiCs incubated under standard and low temperature conditions by Seahorse analysis. Oligomycin, FCCP (carbonyl cyanide-4-(trifluoromethoxy) phenylhydrazone) and rotenone/antimycin were added at the indicated time points (*n* = 3, respectively). Data were analyzed by one-way ANOVA followed by the Mann–Whitney *U* test or Turkey-Kramer method. All data are represented as the mean ± standard error of the mean. *P < 0.05, **P < 0.01, ****P < 0.0001. Cont: control group; Hyp: hypothermia group

We next examined if FFA uptake in the renal tubules can be induced by low temperature itself or if passive uptake occurs from the increased FFAs in the blood. To analyze the direct effects of low temperature on renal tubular cells, we analyzed gene expression changes in the primary cultured mouse renal proximal tubular epithelial cells, MRPTEpiCs, when cultured at low temperatures (Fig. 5A). GSEA showed that the mitochondrial activity-related genes were strongly expressed in the cells exposed to low temperature (Fig. 5B, C, Supplemental Fig. 3). Detoxification-related gene expression was also increased in these cells, suggesting resistance to stress. The RNA-seq analysis showed that *Slc27a1* mRNA expression levels tended to increase in MRPTEpiCs exposed to low temperature, which was confirmed by real-time RT-PCR analysis (Fig. 5D). Immunofluorescence staining revealed abundant expression of FATP1 protein in MRPTEpiCs exposed to low temperature (Fig. 5E). Western blot analysis of protein extracts from similarly treated cells also showed increased FATP1 expression levels at low temperatures (Fig. 5F). Next, palmitic acid (PA) was added to the MRPTEpiC culture medium to mimic high blood FFA levels, followed by lipid accumulation analysis by BODIPY staining to determine if upregulation of *Slc27a1* mRNA expression levels is involved in FFA uptake. Although clear lipid accumulation was observed in the presence of high PA concentrations in low-temperature cultures, interestingly, no intracellular lipid accumulation was observed with high PA culture medium concentrations at 37 °C (Fig. 5G/H). This suggested that the low temperature itself increased FATP1 expression in renal tubular cells and was the initiator of intracellular lipid accumulation. We then analyzed if the FFAs in the low-temperature environment were used as substrates for β-oxidation. The oxygen consumption rate (OCR) of MRPTEpiCs cultured at low temperatures in the presence of PA was determined by Seahorse analysis. Compared with the MRPTEpiCs cultured with PA at 37 °C, these cells cultured with PA at 28 °C showed higher basal and maximal OCRs (Fig. 5I). These results demonstrated that in the mouse proximal tubules, low temperature stimulation itself could induce FFA uptake and an active mitochondrial state, efficiently converting the incoming FFAs into energy by β-oxidation. Furthermore, low temperatures can induce conditions that can relieve the toxicity caused by lipid peroxides from excess FFAs.

### Roles of renal tubular cells and FATP1 expression in hypothermia

**Fig. 6:**
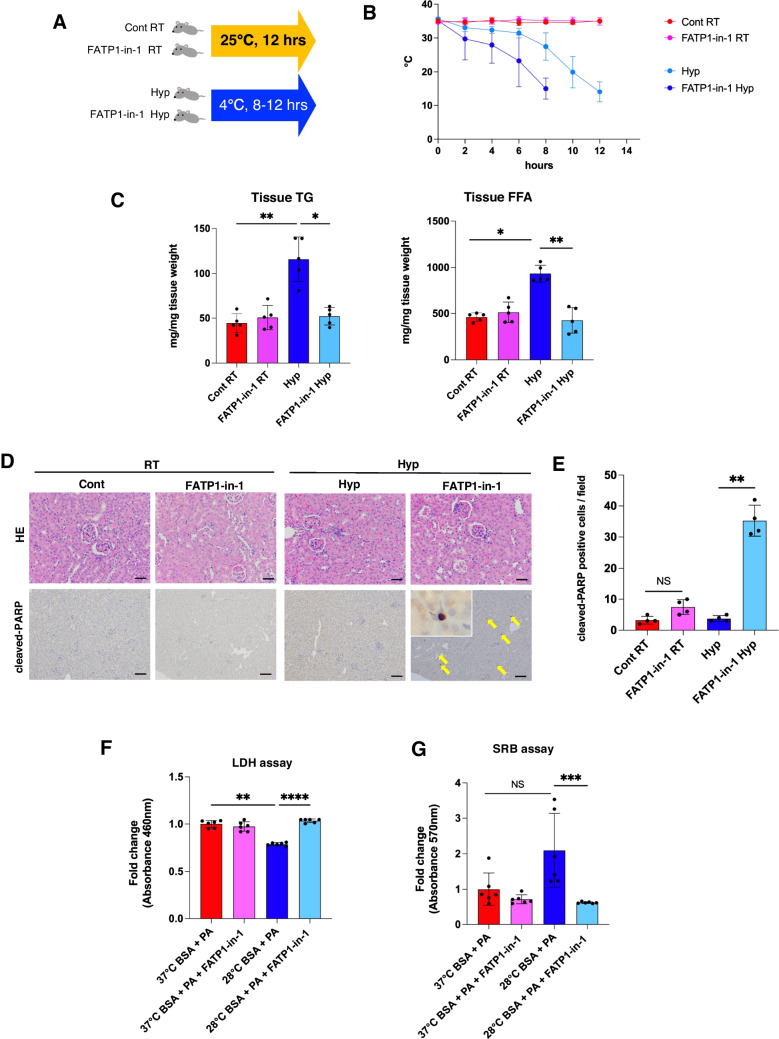
Effect of FATP1 suppression or renal injury on hypothermia. **A** Schematic depiction of the experimental design. RT: kept at room temperature; Hyp: kept at 4 °C; FATP1-in-1: mice treated with 20 mg/kg FATP1-in-1. **B** Changes in body temperature in FATP1-inhibited model mice (*n* = 5 each). **C** FFA and TG levels in renal tissue extracts from the model mice (*n* = 5 each). **D** Hematoxylin and eosin (H&E) staining and immunohistochemical staining of cleaved-PARP in the model mice. Scale bars: 50 μm. **E** The number of cleaved-PARP-positive cells in renal proximal tubular cells. The average of five fields of view at high magnification (× 400). **F** Cytotoxicity LDH assay and SRB assay in mouse renal proximal tubular epithelial cells (MRPTEpiCs) incubated under standard (37 °C) and low-temperature (28 °C) conditions with palmitic acid (PA) and FATP1-inhibitor (*n* = 6). Data were analyzed by the Mann–Whitney *U* test or one-way ANOVA followed by the Turkey-Kramer method. All data are represented as the mean ± standard error of the mean. *P < 0.05, **P < 0.01, ***P < 0.001, ****P < 0.0001. Cont: control group; Hyp: hypothermia group; FATP1-in-1 RT: FATP1 inhibitor room temperature group; FATP1-in-1 Hyp: FATP1 inhibitor hypothermia group

Our analysis suggested that FATP1 plays a central role in activating hypothermia-induced renal tubular β-oxidation. To analyze the significance of FATP1 in hypothermia, we administered FATP1 inhibitors to hypothermia model mice in which tubular cell injury was induced (Fig. 6A). Mice treated with FATP1-in-1 and exposed to hypothermic stress began to display a drop in body temperature 2 h earlier than the hypothermic mice not treated with FATP1-in-1 (Fig. 6B). The FFA and TG levels were quantified in the kidney tissues of these mice, which were lower in the kidneys of the hypothermia model mice treated with FATP1-in-1 than in the hypothermia model mice (Fig. 6C). Hematoxylin and eosin (H&E) staining indicated that the kidneys of neither control mice, nor mice maintained at room temperature, nor mice kept at low temperature after administration of FATP1-in-1 showed obvious dama (Fig. 6D). In contrast, cleaved-PARP-positive cells were observed in the kidneys of hypothermic mice treated with FATP1-in-1, unlike the kidneys of untreated hypothermic mice (Fig. 6D, E). To further investigate if the presence of FFAs and FATP1 is involved in tubular cell survival when exposed to low temperature, we performed an experiment using MRPTEpiC culture. After adding PA and/or FATP1-in-1 to the culture medium, cell membrane damage was evaluated by lactate dehydrogenase (LDH) enzyme activity measurements. The results showed that the leaked LDH activity was significantly reduced in low-temperature cultures with the addition of PA compared with control cultures incubated at 37 °C (Fig. 6F). However, LDH activity increased back to control level when FATP1-in-1 was added to low-temperature culture conditions (Fig. 6F). Furthermore, SRB assays were performed to confirm the survival of cultured cells grown at low temperatures with the addition of PA and/or FATP1-in-1 to the culture medium. In line with the LDH assay results, these experiments revealed higher cell proliferation rates in low-temperature cultures with the addition of PA (Fig. 6G). These results indicated that tubular epithelial cells and their FATP1-mediated FFA uptake are crucially involved in cell survival during hypothermia.

## Discussion

When exposed to a cold environment, homeothermic animals maintain their body temperature using endogenous heat production mechanisms involving involuntary muscle contractions, such as by shivering and non-shivering, to maintain a constant deep body temperature [35–37]. In non-shivering heat production, BAT is known to contribute to thermoregulation by releasing FFAs as an energy source [19, 36, 38]. Forensic autopsies frequently report lipid droplet formation in the proximal tubules of the kidney during hypothermia. However, the mechanism, lipid type, and biological significance of this accumulation remained unclear. In this study, lipidomics analysis was conducted on samples from hypothermia patients and mouse models to determine the pathways of lipid accumulation in the proximal tubules and their biological significance.

Lipidomics analysis of blood samples showed that FFA and FFA-derived product levels were elevated in both the human hypothermia patients and mouse model of hypothermia. These findings suggest that the mouse model we used closely mimics human hypothermia. However, elevated cholesterol levels were observed in human hypothermia, but not in the mouse hypothermia model. Because blood cholesterol originates more from liver synthesis than from dietary sources, the metabolic function of the liver may also be altered in human hypothermia. Increased blood FFA levels also reportedly occur during fasting [39]. Indeed, there are similarities between the lipidomics data from previous analyses in the plasma lipid content of starved people and our data in the present study. However, these data on starvation are the result of 23 to 25 days of fasting. The hypothermia samples we analyzed were cases of accidental hypothermia that occurred within a period of only 12 h. Collectively, these findings suggest that during hypothermia, starvation-like conditions occur rapidly over a period of several hours.

The FFA increase during hypothermia was presumably from non-shivering heat production, with BAT converting fat energy into heat, as previously reported [19, 36]. Moreover, lipid staining demonstrated a notably higher presence of lipid droplets in the proximal tubules of the kidneys in mice exposed to hypothermia compared with the control mice. These results suggest that FFAs in the blood may be incorporated into the proximal tubules by exposure to low temperature. The proximal tubules play an important role in the reabsorption of solutes, such as carbohydrates, amino acids, and electrolytes, filtered by the glomerulus. This reabsorption is highly ATP-dependent. FFAs are therefore recognized as the primary energy source for the proximal tubular cells [28]. FFAs are taken up from the extracellular environment, pass through the cell membrane, and are converted to CoA thioesters, which are then used for subsequent β-oxidation. Extracellular FFA uptake is reportedly facilitated by CD36 and the FATP/Slc27 protein family [40–43]. Our RNA-seq analysis revealed upregulated *Slc27a1* mRNA expression levels in hypothermia model renal tissues compared with those of normal controls. Immunohistochemistry assays showed high FATP1 protein expression patterns in the proximal tubules. However, hypothermia did not increase CD36 expression at the mRNA or protein level. Furthermore, we observed that the low temperature stimulus itself could increase FATP1 expression in renal proximal epithelial cell culture experiments. These results suggested that hypothermic stimulation dominantly increases FATP1 expression levels in the renal proximal tubules, resulting in increased FFA uptake from the blood. In the present study, we found that *Plin5* mRNA and its corresponding protein were strongly expressed in hypothermic mice renal tissues. PLIN5 protein coats the surface of intracellular lipid droplets and has been shown to play a role in regulating the conversion of FFAs to TGs, a process known as lipogenesis [33, 44, 45]. PLIN5 can promote the formation of TGs by interacting with enzymes involved in lipogenesis, such as diacylglycerol acyltransferases (DGATs). In addition, PLIN5 on the surface of lipid droplets can interact with mitochondria and stimulate FFA uptake and transport into the mitochondria for β-oxidation in various cell types [28, 46]. Our TEM analysis demonstrated that lipid droplets were in contact with renal tissue mitochondria in the hypothermia model. Upregulated mRNA levels of *Slc27a1*, *Plin5*, and *Cpt1a*, genes that are associated with lipid metabolism, were observed in the hypothermic renal tissues. Increased expression patterns of their respective proteins were also noted, suggesting an augmentation of β-oxidation in these renal tissues. Expression of these transcripts is known to be regulated by PPARα [47–49], which was also upregulated in the renal tissues of the hypothermia model according to our RNA-seq and Western blot analyses. Immunohistochemical staining demonstrated positive staining of the proximal tubule nuclei in the hypothermia model renal tissues, indicating that PPARα was transcriptionally active. PPARα-mediated transcription is known to be activated by the binding of FFAs that were taken up by the cell [48, 50]. Interestingly, low temperature conditions could enhance the expression levels of *Slc27a1* mRNA, but not those of *Ppara* mRNA, in primary cultured mouse tubular cells. Taken together, our findings suggest that the low temperature itself increases FATP1 expression levels in the hypothermic renal tubules, resulting in intracellular FFA uptake. This thereby activates the transcriptional function of PPARα, increases PLIN5 and CPT1A expression, and activates β-oxidation. However, the specific mechanisms by which low temperature itself can increase FATP1 mRNA and protein levels require further investigation. Because the expression of so-called “cold shock” proteins, such as cold-inducible mRNA binding protein (CIRBP), is enhanced in hypothermic renal tissues and cultured tubular cells in low temperature conditions, it would be worth noting if these proteins are involved in regulating FATP1 expression.

Lipid accumulation in the renal tubules is thought to be caused by a chronic increase in blood FFAs associated with abnormal lipid metabolism [25, 28, 48, 49, 51–53]. We investigated if the lipid accumulation in renal tubules during hypothermia is passively induced by a rapid increase in blood FFA concentration over a short period. In experiments with cultured mouse tubular epithelial cells, we observed that even in the absence of PA in the medium, low temperatures alone could increase FATP1 expression levels. This resulted in lipid accumulation and enhanced β-oxidation within 5 h of PA addition. However, intracellular lipid accumulation did not occur when PA was added to the medium at 37 °C culture conditions, which did not induce FATP1 expression. These findings indicate that renal tubular cells rapidly and vigorously take up FFAs, obtaining energy through β-oxidation under hypothermia in normal oxygen conditions. Excessive levels of FFAs can be toxic to cells, especially when accumulated in non-adipose tissues [54–56]. After FFAs are taken up, the mitochondria rapidly use a portion of them as substrates for β-oxidation. However, the excess FFAs are converted to TGs, which are not toxic to cells in lipid droplets [24, 48, 49, 52, 53, 57, 58]. This implies that FFAs are minimally toxic to cells if they are quickly converted into a substrate for β-oxidation or stored as TGs. GSEA of renal tissue samples from the mouse hypothermia model did not show gene sets that would indicate obvious renal damage. In addition, histopathological analysis of these renal tissues also showed no obvious damage. Therefore, the kidneys appear to appropriately process lipids during hypothermia, but the biological significance of activated β-oxidation in the renal tubules during hypothermia remains in question. To clarify this, we examined FATP1 inhibitor-treated mice to limit lipid uptake. The mice with inhibited lipid uptake into the renal tubules exposed to low temperatures showed a rapid decrease in body temperature. Furthermore, FATP1 inhibition induced cell death in renal tubules under hypothermic conditions. These findings suggest that maintaining proper renal lipid metabolism may play a crucial role in the response to cold stress.

In conclusion, our work suggests that low temperature can increase renal proximal tubular FATP1 expression levels and FFA uptake, which in turn promotes β-oxidation. Preservation of this kidney function during rewarming therapy for hypothermia may have a positive impact on patient prognosis.

Supplementary information.

## Supplementary Information

Below is the link to the electronic supplementary material.

ESM 1 (XLSX 14.2 MB)

ESM 2 (PPTX 5.08 MB)

## Data Availability

After acceptance of the paper, we upload and release the RNA-seq data.
